# Stroboscopic thermally-driven mechanical motion

**DOI:** 10.1038/s41598-022-24074-z

**Published:** 2022-11-22

**Authors:** Luca Ornigotti, Radim Filip

**Affiliations:** 1 Department of Optics, Palacký University, 17. listopadu 1192/12, 771 46 Olomouc, Czech Republic; 2 Quantum Optics, Quantum Nanophysics and Quantum Information, Faculty of Physics, University of Vienna, Boltzmanngasse 5, 1090 Wien, Austria

**Keywords:** Optics and photonics, Optical physics, Condensed-matter physics, Statistical physics, thermodynamics and nonlinear dynamics

## Abstract

Unstable nonlinear systems can produce a large displacement driven by a small thermal initial noise. Such inherently nonlinear phenomena are stimulating in stochastic physics, thermodynamics, and in the future even in quantum physics. In one-dimensional mechanical instabilities, recently made available in optical levitation, the rapidly increasing noise accompanying the unstable motion reduces a displacement signal already in its detection. It limits the signal-to-noise ratio for upcoming experiments, thus constraining the observation of such essential nonlinear phenomena and their further exploitation. An extension to a two-dimensional unstable dynamics helps to separate the desired displacement from the noisy nonlinear driver to two independent variables. It overcomes the limitation upon observability, thus enabling further exploitation. However, the nonlinear driver remains unstable and rapidly gets noisy. It calls for a challenging high-order potential to confine the driver dynamics and rectify the noise. Instead, we propose and analyse a feasible stroboscopically-cooled driver that provides the desired detectable motion with sufficiently high signal-to-noise ratio. Fast and deep cooling, together with a rapid change of the driver stiffness, are required to reach it. However, they have recently become available in levitating optomechanics. Therefore, our analysis finally opens the road to experimental investigation of thermally-driven motion in nonlinear systems, its thermodynamical analysis, and future quantum extensions.

## Introduction

Levitating optomechanics has rapidly developed during the last decade, combining the fast and precise classical optical control of nanoparticle motion with sensitive high-speed and shot-noise-limited optical detection^1–7^. The critical achievements are fast and deep cavity cooling^8–19^ and feedback cooling^20–24^ near the quantum mechanical ground state. Furthermore, potential shaping towards tilted double-well^25–27^, local cubic^28,29^, and quartic potentials^30^ has allowed to observe a plethora of nonlinear phenomena. Despite the already deep vacuum, the particle feels the hot environment from the trapping beam, and thermalisation forces the experiments towards attractive and unexplored transient dynamics. In parallel, theoretical proposals set attractive goals for classical and quantum nanoscale mechanical engines^31–33^, and even the realisation of macroscopic quantum superpositions^34–37^. Simultaneously, particle levitation and cooling in 2D and 3D have been developed^38–40^, and the interaction of levitating particles is currently being investigated^41–43^. It opens a larger space to observe transients of interacting systems^44^ and, possibly, even complex systems^45,46^.

Levitating optomechanics has opened an extensive theoretical and experimental investigation of transient, unstable motion in nonlinear potentials^47–50^. A pivotal nonlinear effect uses fluctuations positively to generate directional motion^29,51,52^. It is an autonomous motion that does not require an external coherent drive. It has been experimentally verified in the overdamped regime^29,51^, and recently investigated in the underdamped limit^52^. However, such phenomena are limited when the directional motion and the fluctuating driver are combined in one degree of freedom (1D). Using a nonlinear rectifying potential (like $x^4$) to reduce noise comes with a nontrivial experimental challenge that requires a particle strongly diverging from the instability to be sufficiently slowed down. Additionally, a linear or nonlinear dissipative control can be destructive, especially in the quantum regime. Lastly, measurement back-action precludes any feedback control from being used as an alternative motion rectification strategy in the quantum regime.

An unexplored motion in a 2D nonlinear potential, or equivalently a nonlinear interaction of two particles, potentially promises to break such limitations and allow further explorations. It aims at generating displacement in a part of the system (position x) via a nonlinear coupling with the noisy degree (position y) without a negative back-action of *x* to *y*. However, *y* remains unstable, and the increased noise has to be rectified to keep the experiment under control.

This paper proposes and analyses the stroboscopic cooling and confinement of the noisy driver. It aims at reaching an autonomous and less diverging thermally induced mean displacement of the system, growing faster than its standard deviation. For simplicity of analysis, we focus on a minimal model for unstable dynamics. It incorporates the nonlinear force exerted on *x* by another fluctuating degree of freedom *y*, and their respective surrounding environment that are considered out of equilibrium between the two directions *x*,*y*. Such 2D potential arrangements are feasible, although unexplored, for nanoparticles in current optical traps. They allow the possibility to separate the directional motion in *x* and the noisy driver in *y*, and better exploit their complementary roles. The minimal model is based on a 2D potential $V_D(x,y)\propto xy^2$ or, alternatively, two-particle system.

The model and analysis are organised as follows. We consider an out-of-equilibrium dynamics, where the signal position *x* is coupled to a low-temperature bath at $T_x$, whereas the noisy driver *y* couples to a high-temperature bath $T_y$. For any damping, a position uncertainty in *y* drives the position change in *x* at the cost of a back action ($x\rightarrow y$), changing the stiffness of *y*. In turn, the mean displacement $\langle x \rangle$ becomes more negative, and its action on *y* introduces instability. It increases the negative effective quadratic stiffness in the driver *y*. The first step is turning it into an efficient nonlinear thermal drive is to avoid the negative back-action effect, which limits the performance as the instability leads to noise explosion in *y*, and subsequently, in *x*.

We assume the particle with mass *m* and the trajectories *x*, *y* to be initially centered at $\langle x_0 \rangle, \langle y_0\rangle =0$, with an uncertainty $\sigma _{x_0}^2=k_B T_{0,x}/m\omega _{0,x}^2$, and $\sigma _{y_0}^2=k_B T_{0,y}/m\omega _{0,y}^2$. Such initial conditions are obtained by a particle pre-cooling step in a harmonic potential with stiffnesses $\omega _{0,x}^2,\omega _{0,y}^2$, where $T_{0,x},T_{0,y}$ are effective initial temperatures of *x* and *y*, with $k_B$ being the Boltzmann constant. At time $t=0$ we switch this harmonic 2D potential to a nonlinear 2D potential $V_D(x,y)$.

To stimulate the majority of the current optical levitating experiments, by reducing requirements for fast detection, the most basic model describes the dynamics with $V_D(x,y)$ in the overdamped limit by the Langevin equations

1 $$\begin{aligned} \dot{x} = - k y^2 + \sqrt{\frac{2k_B T_x}{\Gamma }}\xi _x(t),\qquad \dot{y} = -2 k x y +\sqrt{\frac{2k_B T_y}{\Gamma }}\xi _y(t), \end{aligned}$$

where $k=\kappa /\Gamma$ is the normalised strength of the interaction to the damping coefficient $\Gamma$. The stochastic forces $\xi _i$, describing separated baths for the *x* and *y* directions, are independent, delta-correlated, zero-mean Gaussian white Markovian noise, $\langle \xi _i(t_1)\xi _i(t_2) \rangle =\delta (t_1-t_2)$, $\langle \xi (t) \rangle =0$ ^53^. Their magnitude is controlled by the respective diffusion coefficients $D_x= 2 k_B T_x/\Gamma$ and $D_y= 2 k_B T_y/\Gamma$, characterised by the drag coefficient of the medium $\Gamma$, equal for both *x* and *y*. Temperatures $T_x$ and $T_y$ characterise the separated baths. For *y* variable, we define a cooling depth $C=T_{y,0}/T_y$, $T_{y,0}<T_y$, at initial time for further use.

In Eq. (1, left), the coupling term $-k y^2$, $k>0$, quadratically depends on the position of another degree of freedom *y* evolving in Eq. (1, right). This term acts on *x* as a fluctuating force if a back-action of *x* to *y* is negligible. Its nonlinear form implies that fluctuations of *y* translate into the systematic shift of the mean value of *x*, and its fluctuations. The first term in Eq. (1, right) $-2kxy$ is, therefore, unwanted back-action effect, changing the stiffness of the driving variable *y* and introducing more unstable inverted-quadratic potential in *y* for increasing negative signal variable *x*.

We quantify the position shift of *x* via the mean displacement signal $\langle x \rangle$, and signal to noise ratio $\textrm{SNR} = |\langle x \rangle | /\sigma _x$, where $\sigma _x$ is the standard deviation of position *x*. A better performance means obtaining a higher $\textrm{SNR}$ for the same mean displacement signal. Note, $\textrm{SNR}$ is very sensitive to unstable dynamics^29,51^, therefore it improves if for the same $\langle x\rangle$ the standard deviation $\sigma _x$ is less increasing. The sufficient threshold for an observable is $\textrm{SNR}=1$, as it corresponds to the conventional Rayleigh resolution limit for displaced distribution maxima, assuming ideal particle detection^54,55^.

We show that in the limit of a significant *temperature bias* $T_x\ll T_y$ we can reasonably approximate the thermally induced effect by one-way driving of the signal *x* by quadratic $y^2$ position. Numerical simulations of Eq. (1) in the above regime produce a signal-to-noise ratio threshold of $\sqrt{3}/2$; the bound that will be retrieved analytically after. However, the dynamics in *y* remains unstable and noisy. It limits the detection of the nonlinear effect as the rapidly rising instability, and noise in the displacement *x* makes its signal-to-noise drop. Consequently, it restricts further developments and applications. A higher-order rectifying nonlinearity, such as $V_R(y)\propto y^4$, is required to reduce such noise explosion in *x*. However, its implementation in optical trapping is generally challenging, especially for an extensive range of *y*. We, therefore, suggest a viable and advantageously still autonomous approach, a fast and deep *stroboscopic cooling*. It requires a periodic stiff confinement of *y*, aimed at re-initiating its state while the variable *x* evolves continuously. Importantly, this autonomous method does not need the measurement of either *x* or *y*, as the speed and depth of cooling are optimised for the parameters of Eq. (1). It allows to extend the methodology to lower pressures and temperatures, into the quantum regime. We analyse the stroboscopic thermally-induced motion and demonstrate that it can reach a large signal-to-noise ratio at a reasonable cost of increasing speed and depth of cooling.

## Results

### Single cycle overdamped dynamics

**Figure 1:**
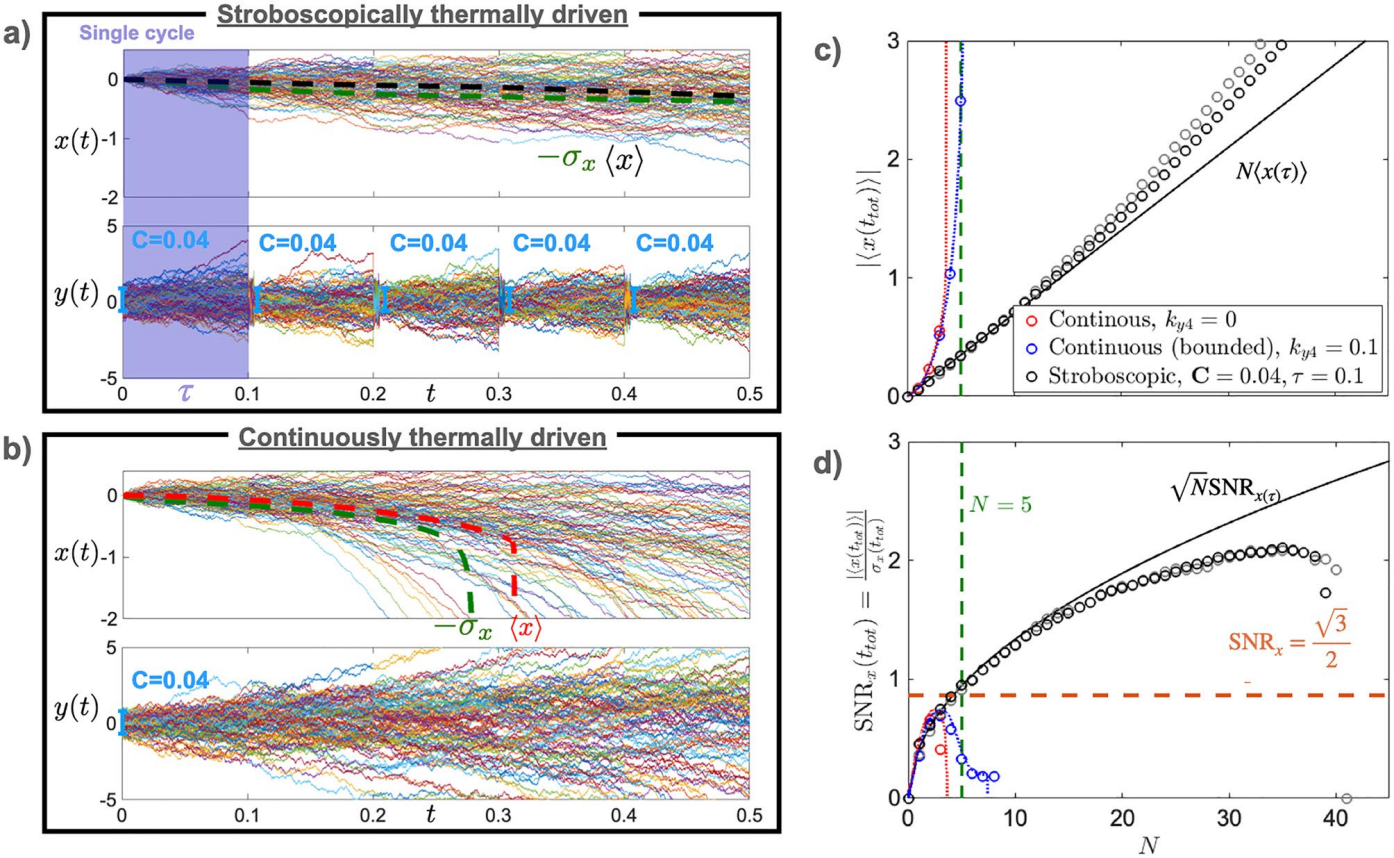
Thermally-driven motion in *x* with stroboscopic cooling of *y* variable compared to continuous dynamics in *y* without the cooling. (**a**) At every repetition, after an interval $\tau$, the noisy driver *y* is fastly and stroboscopically cooled in a confined potential to a depth of cooling $\textbf{C}=T_{0,y}/T_y$. On the other hand, the signal variable *x* evolves continuously. Mean position (dashed black) and standard deviation $\sigma _x$ (dashed green) grow comparably, reaching the signal-to-noise ratio $\textrm{SNR}_x=1$ already after $N \approx 6$ repetitions. (**b**) Both the signal *x* and the driver *y* are evolving without any stroboscopic cooling. Standard deviation (dashed green) grows faster than mean position (dashed red) resulting in a drop of the $\textrm{SNR}_x$. In both cases (a,b) the y-trajectories are initially cooled to $\sigma _{y_0}^2=k_BT_{0,y}/m\omega _0^2=0.2$. (**c**, **d**) Comparison of the approaches (**a**) and (**b**), by means of mean displacement signal $\langle x(t_{tot}) \rangle$ (**c**), and signal-to-noise ratio $\textrm{SNR}_x(t_{tot})$ (d) over $N=t_{tot}/\tau$ time intervals. The $\textrm{SNR}_x(t_{tot})$ (red, blue) obtained from panel (**b**) quickly drops ($N \approx 3$), without reaching the bound $\sqrt{3}/2$ (orange). Blue dots are obtained by adding a quartic bound to the driver *y*, for a comparison. In contrast, the $\textrm{SNR}_x(t_{tot})$ (black) obtained from panel (**a**) climbs above the limit of single cycle dynamics (orange), and the critical value $\textrm{SNR}_x=1$ in $N\approx 5$. It grows thrice as large as the $\textrm{SNR}_x$ obtained from continuous dynamics before it drops. Note: the grey dots are obtained by assuming the re-initialisation of the driver *y*, instead of the cooling. It is shown to justify the analytical approximation used in Eq. (5) (solid black).

To develop the stroboscopic control of a nonlinear mechanical oscillator, we must first understand its single cycle dynamics and attain it close to a one-way interaction where the back-action can be neglected.

To describe such a regime of pure nonlinear drive, we require the back-action term 2*kxy* to be negligible to the thermalisation Langevin forces in *y*. Furthermore, we first assume $\langle x_0 \rangle, \sigma _{x_0}^2=0$, such that the integration of Eq. (1) yields to the evolution of the signal variable $x(t) = - k \int _0^t y^2(t')dt' + \sqrt{2k_BT_x/\Gamma } \int _0^t \xi _x(t')dt'$. The mean evolution $\langle x(t) \rangle = -k\int _0^t \langle y^2(t') \rangle dt'$ is driven by the autocorrelation of the drive variable $\langle y^2(t') \rangle =\langle y_0^2\rangle + 2 \langle y_0\rangle \sqrt{2 k_B T_y/\Gamma } \int _0^{t'} \langle \xi _y(t_1)\rangle dt_1 + 2k_B T_y/\Gamma \iint _0^{t'} \langle \xi _y(t_1)\xi _y(t_2)\rangle dt_1\,dt_2=\sigma _{y_0}^2 + 2 k_B T_y t' /\Gamma$. With the assumption of $T_x=0$, the second moment $\langle x^2(t) \rangle =k^2\iint _0^t \langle y^2(t')y^2(t'')\rangle dt'\,dt''$ depends solely on the higher order correlation $\langle y^2(t')y^2(t'')\rangle$ the full derivation of which is obtained in the Methods Section “[Sec Sec8]”.

All equations thereafter are calculated considering $k_B=1\left[ \textrm{m}^2 \textrm{Kg}/ \textrm{s}^2 \textrm{K} \right]$, $m=1 \left[ \textrm{Kg}\right]$, $\Gamma =1 \left[ \textrm{Kg}/\textrm{s} \right]$, $\omega _{0,y}^2,\omega _{0,x}^2=1 \left[ 1/ \textrm{s}^2\right]$.

As a result, the moments of the signal variable *x* evolve during the first cycle $\tau$ as follows

2 $$\begin{aligned} \langle x(t) \rangle = -k T_y \left( \frac{\textbf{C}}{t} + 1\right) t^2, \qquad \sigma _{x(t)} = \frac{2}{\sqrt{3}}k T_y \left( \frac{3 \textbf{C}^2}{2 t^2} +2\frac{\textbf{C}}{t} +1\right) ^{\frac{1}{2}} t^2,\qquad \textrm{SNR}_{x(t)} =\frac{| \langle x \rangle |}{\sigma _x} = \frac{\sqrt{3}}{2} \frac{\frac{\textbf{C}}{t} + 1}{\sqrt{\frac{3\textbf{C}^2}{2t^2} +2\frac{\textbf{C}}{t} +1}}. \end{aligned}$$

Note that at initial time $t=0$ the drive *y* is prepared by cooling at given $\textbf{C}$ to generate the initial variance $\sigma _{y_0}^2$. The depth of cooling $\textbf{C} = [0,1]$ is lower bounded by zero (full-depth cooling $T_{0,y}=0$), and upper bounded by one (continuous dynamics $T_{0,y}=T_y$).

All the figures in this manuscript are generated by numerical solution of Eq. (1). The initial conditions are described by a Gaussian distribution centered in $\langle x_0 \rangle,\langle y_0 \rangle =0$, with a standard deviation $\sigma _{x_0}^2=0$,$\sigma _{y_0}^2=0.2$. Unless stated otherwise, the interaction strength $k=1$, environmental temperatures $T_x=0.1,T_y=5$, and timestep $dt=6.6 \times 10^{-5}$ are employed to produce $10^4$ trajectories with an ensemble size of 1500 for each repetition *N*. Furthermore, the depth of cooling $\textbf{C}=0.04$ periodically leads to the variance $\sigma ^2_y=0.2$.

In the limit of $\textbf{C}/t \rightarrow 0$ the signal-to-noise ratio of Eq. (2) reaches the threshold value of $\sqrt{3}/2$, reported in Fig. 1 (orange dashed panel d). Such limit is obtainable by either long time continuous dynamics $t\rightarrow \infty$, $\textbf{C}=1$, or by full-depth cooling $\textbf{C} \rightarrow 0$ for any time *t*. However, as time increases, the back-action $-kxy$ introduces instabilities in the drive variable *y* (Fig. 1 panel b), which in turns makes the noise $\sigma _x$ (dashed dark green) grow faster than the mean position $\langle x \rangle$ (dashed red). Ultimately it hinders the position *x* from reaching $\textrm{SNR}_x=\sqrt{3}/2$ (Fig. 1, blue and red dots, panel d), thus limiting its dynamics to short transients (about $3\tau$) before observing the drop of the signal-to-noise ratio. Altogether, $\textrm{SNR}_x$ cannot go beyond unity, which limits the observability of the effects.

In the case of deviations from vanishing $T_x$, and $\sigma _{x_0}^2$, for $\langle x_0 \rangle =0$, the evolution of moments of particle position *x* of Eq. (2) becomes

3 $$\begin{aligned} \begin{aligned} \langle x(t) \rangle&= -k T_y \left( \frac{\textbf{C}}{t} + 1\right) t^2,\quad \sigma _{x(t)} = \frac{2}{\sqrt{3}}k T_y \left( \frac{3 \textbf{C}^2}{2 t^2} +2\frac{\textbf{C}}{t} +1\right) ^{\frac{1}{2}}\left[ 1+ \theta \right] ^{\frac{1}{2}}t^2,\quad \textrm{SNR}_{x(t)} =\frac{| \langle x \rangle |}{\sigma _x} =\frac{\sqrt{3}}{2}\frac{\frac{\textbf{C}}{t} + 1}{\sqrt{\frac{3\textbf{C}^2}{2t^2} +2\frac{\textbf{C}}{t} +1}\sqrt{1 + \mathbf {\theta }}},\\ \theta&= \theta _0(\textbf{C},t,\sigma _{x_0}^2) + \theta _E(\textbf{C},t,T_x), \quad \theta _0=3\sigma _{x_0}^2\frac{(3\frac{\textbf{C}^2}{2t^2} +\frac{2\textbf{C}}{t} +1)^{-1}}{4k^2T_y^2t^4}, \quad \theta _E=3T_x\frac{(\frac{3\textbf{C}^2}{2t^2} +\frac{2\textbf{C}}{t} +1)^{-1}}{2k^2T_y^2t^3}, \end{aligned} \end{aligned}$$

where $\theta$ describes the effects of the initial position uncertainty $\sigma _{x_0}^2$ via $\theta _0$, and environmental noise $T_x$ through $\theta _E$ which decrease the signal-to-noise ratio from $\textrm{SNR}_x=\sqrt{3}/2$ at short times. Note that in the limit of long time dynamics $t \rightarrow \infty$, the signal-to-noise ratio of Eq. (3) still converges to the $\textrm{SNR}_x=\sqrt{3}/2$ bound obtained from Eq. (2), irrespective of $T_x$ and $\sigma _{x_0}^2$. In the limit of full-depth cooling $\textbf{C} \rightarrow 0$, the signal-to-noise ratio approaches $\textrm{SNR}_x \approx \sqrt{3}(1+3(\sigma _{x_0}^2+2T_x t)/4k^2T_y^2t^4)^{-\frac{1}{2}}/2$. To reach $\textrm{SNR}_x=\sqrt{3}/2$, the initial variance $\sigma _{x_0}^2$ and the environmental temperature $T_x$ need to be minimised. The initial variance can be efficiently minimised by initially cooling the motion of position *x* towards $\sigma _{x_0}^2 \rightarrow 0$^7^. The environmental noise dictated by $T_x$, large for the cryogenic temperatures, can be minimised by a temperature bias $T_y \gg T_x$, as follows from $\theta _E$.

For comparison, when approaching the opposite regime of $\textbf{C}/t \gg 1$, obtained for short transients $t \ll 1$ at a given nonvanishing $\textbf{C}$, the signal-to-noise ratio of Eq. (3) approaches $\textrm{SNR}_x \approx (1+ (\sigma _{x_0}^2 + 2T_x t)/2\textbf{C}^2 k^2 T_y^2 t^2)^{-\frac{1}{2}}/\sqrt{2}$. By minimising both the initial variance $\sigma _{x_0}^2$, and the environmental noise $T_x$, a cooling of the driving variable *y* away from the full-depth cooling $\textbf{C}>0$ allows to reach a signal-to-noise ratio close to $\textrm{SNR}_x=\sqrt{3}/2$. Such limitation can be observed in Fig. 1 (panel d, red and blue dots) where the signal-to-noise ratio cannot reach the bound even for near full-depth cooling $\textbf{C} =0.04$ before it drops due to the back-action (we use timescale $t=\tau N$).

### Stroboscopic control

**Figure 2:**
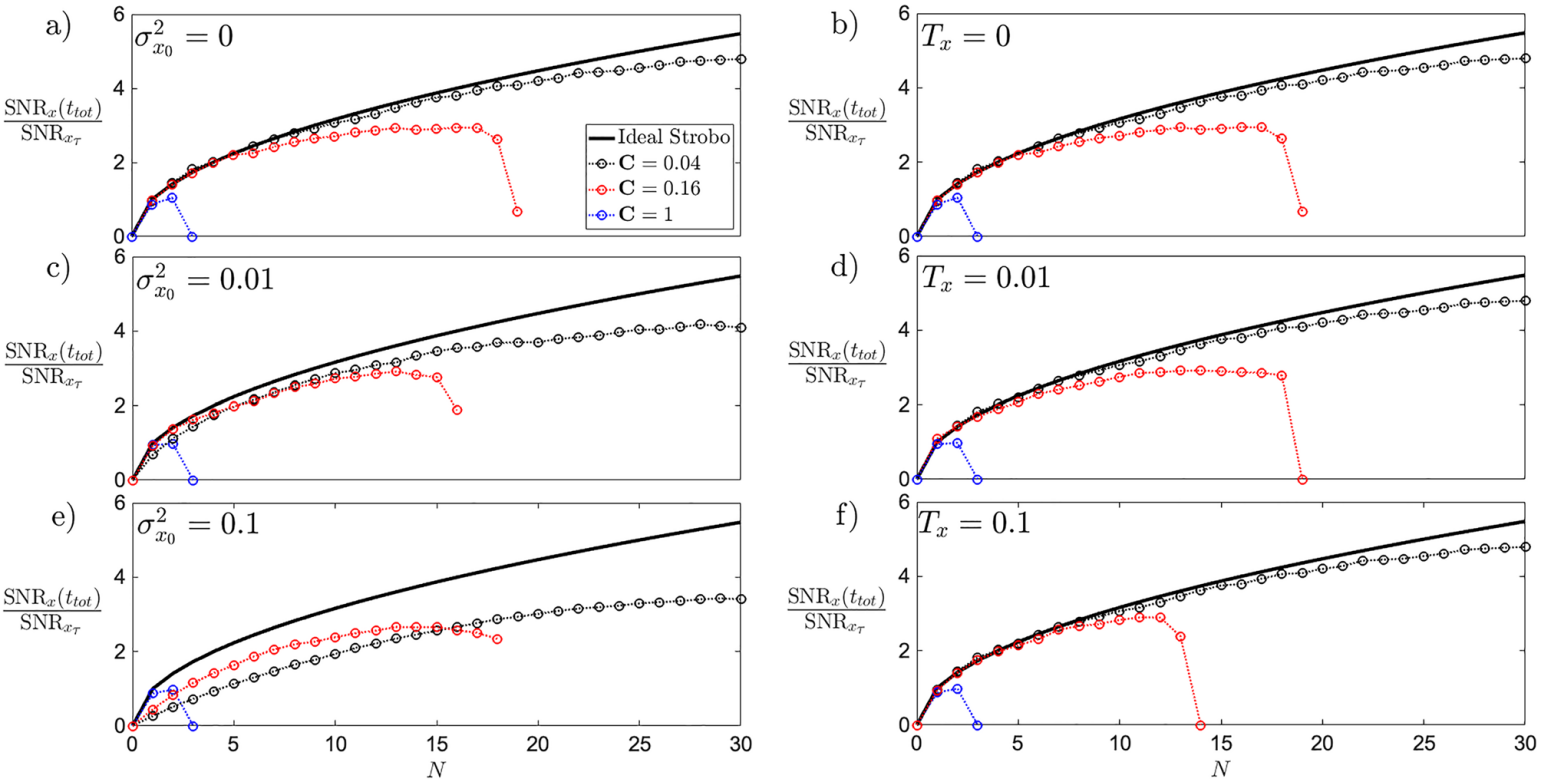
Sensitivity, and robustness of the SNR to initial variance $\sigma _{x_0}^2$ (left) and environmental temperatures $T_x$ (right) in the stroboscopic control. The comparison is generated for different depth of cooling $\textbf{C}$. The solid black line indicates the scaling of the back-action-less evolution of Eq. (5) in the ideal case of $\langle x_0 \rangle, T_x,\sigma _{x_0}^2=0$. Increasing the initial variance (panels **a**,**c**,**e**) results in an overall worsening of the SNR that evolves with a non-ideal scaling regardless of the cooling depth. Oppositely, noisier environments (panels **b**,**d**,**f**) do not affect the scaling, but the amount of repetitions *N* before the drop of the signal-to-noise ratio occurs.

To significantly improve the signal-to-noise ratio over $\textrm{SNR}_x=\sqrt{3}/2$, a stroboscopic control is implemented. It periodically cools and confines the continuous motion of a particle in *y*, by a series of fast cooling events *N* provided by a separated cold bath at a temperature $T_{y,0}$.

With every new repetition, realised after time $\tau$ (time of the single period for $N=1$), the driver *y* is confined in a quadratic potential of strength $\omega _{0,y}^2=1/\textbf{C}$ for a time $t_{C}=\tau /20$. Such confinement, together with cooling, is used to periodically and quickly restore the fluctuations of *y* to its initial variance $\sigma _{y_0}^2=k_B T_{0,y}/m\omega _{0,y}^2$. It is a necessary step to avoid the divergence of the *y*-trajectories.

In the regime of weak coupling $k \le 1$, the correlation of the *y*-trajectories between two stroboscopic steps is negligible. The simultaneous cooling and confining allows to approximately assume a re-initialisation of the driver. However, in the regime of strong coupling $k>1$, the fast growing back-action makes the cooling protocol more challenging. In addition to the previous protocol, it requires the coupling *k* to be switched off during the cooling step. This ultimately allows to efficiently overcome the instability introduced by the back-action, before the fluctuations of the *y*-trajectories can be restored.

Simultaneously, the signal variable *x* continues its dynamics over time $t_{tot}=N\tau$ without being acted upon. Such stroboscopic evolution, as observed in Fig. 1 (panel a), allows the rectification of the motion of *x* with a standard deviation (dashed dark green) growing comparably to its mean (dashed black). Therefore, stroboscopic control substitutes the challenging higher order quartic potential, otherwise needed to control the diverging *y*-trajectories.

The stroboscopic control can be approximately described by $x(t_{tot}) = x_0 - k \sum _{n=0}^{N} \int _0^{\tau }y^2_{n}(t') dt' + \sqrt{2k_BT_x/\Gamma }\int _0^{t_{tot}} \xi _x(t')dt'$, where the term $y_n(t')=y_{0,n} + \sqrt{2k_BT_y/\Gamma }\int _0^{t'}\xi _y(t_1)dt_1$ describes the statistically independent dynamics of the driving variable *y* split in the period $\tau$ between two cooling events (see second section of the Methods “Stroboscopic evolution” for a detailed discussion). See Fig. 1c,d for a justification of this re-initializing approximation (grey dots) in comparison with the full simulation (black dots). To compute higher moments of *x*, we need the auto covariance $\textbf{K}_{n,n}(t',t'')=\langle y^2_n(t') y^2_n(t'') \rangle -\langle y^2_n(t') \rangle \langle y^2_n(t') \rangle$ of $y^2$ within the period $\tau$ (see Methods “Stroboscopic evolution” for the full discussion). The moments of the signal’s variable *x* can be written, for $\langle x_0 \rangle =0$, as

4 $$\begin{aligned} \langle x(t_{tot}) \rangle = k\sum _{n=0}^{N} \int _0^{\tau } \langle y^2_n(t') \rangle dt', \quad \sigma _{x}(t_{tot}) = \sqrt{ \sigma _{x_0}^2 + k^2\sum _{n=0}^N \iint _0^{\tau }\textbf{K}_{n,n}(t',t'')dt' dt'' + \frac{2k_B T_x t_{tot}}{\Gamma }}, \quad \textrm{SNR}_x(t_{tot}) = \frac{| \langle x(t_{tot}) \rangle |}{\sigma _{x}(t_{tot})}. \end{aligned}$$

In the case of ideal control, i.e. back-action-less regime in absence of the $-kxy$ term, and $\sigma _{x_0}=0, T_x=0$, the moments of Eq. (4) increase by a constant amount at each repetition. It simplifies the above to

5 $$\begin{aligned} \langle x(t_{tot}) \rangle = N \langle x(\tau ) \rangle,\qquad \sigma _{x}(t_{tot}) = \sqrt{N} \sigma _{x(\tau )},\qquad \textrm{SNR}_x(t_{tot}) = \sqrt{N} \textrm{SNR}_{x(\tau )}, \end{aligned}$$

where the quantities $\langle x_{\tau } \rangle, \sigma _{x_{\tau }}, \textrm{SNR}_{x_{\tau }}$ are the mean, standard deviation, and signal-to-noise ratio of position *x* calculated at time $\tau$ from Eq. (2).

**Figure 3:**
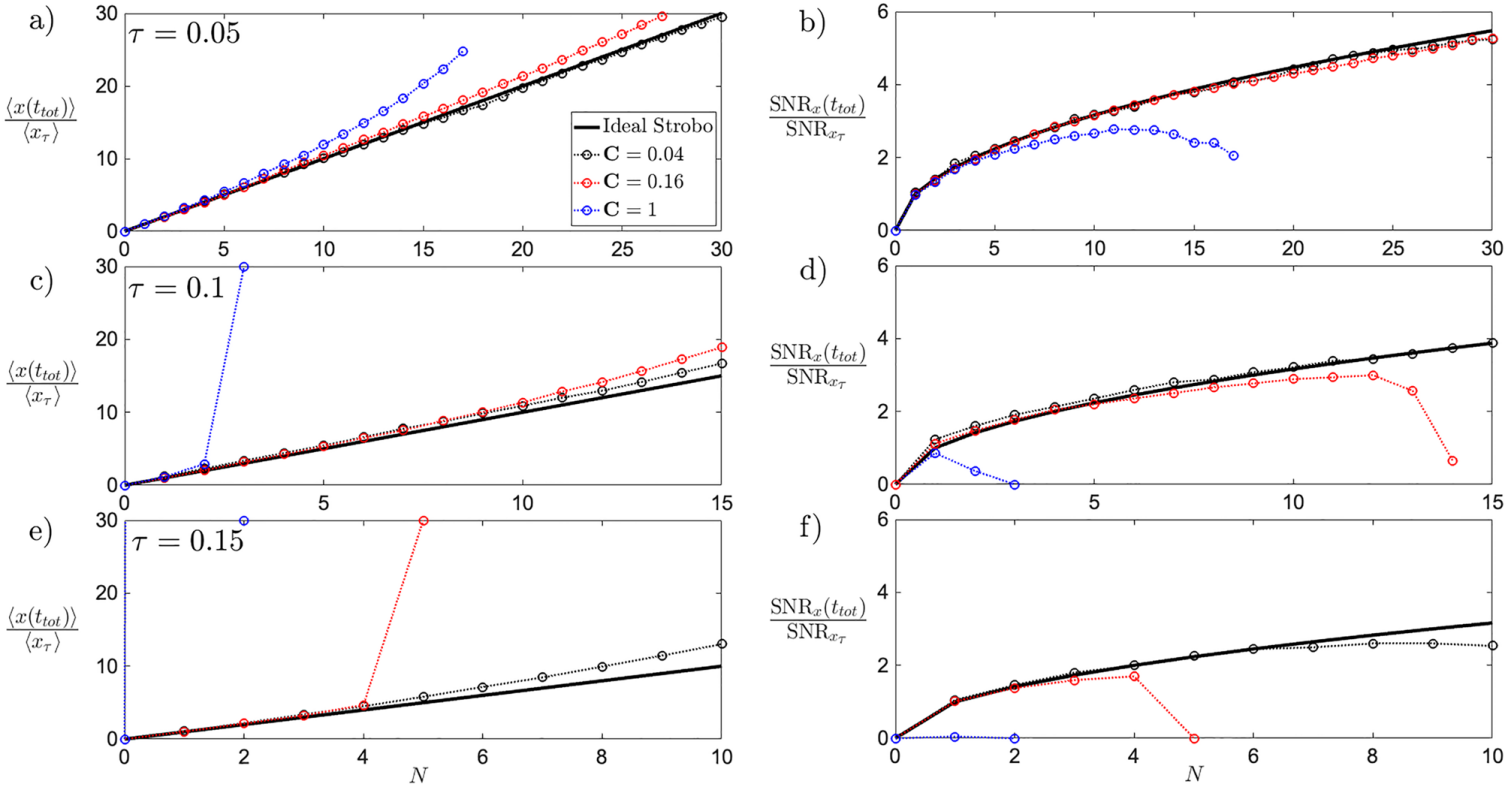
Stroboscopic evolution of mean position $\langle x(t_{tot}) \rangle$ (**a**,**c**,**e**) and $\textrm{SNR}_x(t_{tot})$ (**b**,**d**,**f**) of the system variable *x*, with cooling scheme of the drive variable *y* for different depths of cooling $\textbf{C}$ (dots) at different single cycle times $\tau$. The total time $t_{tot}=N\tau$ is kept constant from panel (**a**) to (**f**), allowing for vertical comparison. The solid black line indicates the scaling of the back-action-less evolution of Eq. (5) in the ideal case of $T_x=0,\sigma _{x_0}^2=0$. As the single cycle time $\tau$ increases, the back-action requires less cooling events *N*, for the same overall time $t_{tot}$ to dominate the dynamics and make the $\langle x(t_{tot}) \rangle$ (**a**,**c**,**e**) deviate from the most ideal case (solid black), while the $\textrm{SNR}_x(t_{tot})$ (**b**,**d**,**f**) drops. The effect is enhanced as $\textbf{C}$ increases (red, blue). Shorter $\tau$ (**a**,**b**), allow to accommodate larger values of $\textbf{C}$ while keeping the dynamics close to a negligible back-action regime. In contrast, larger $\tau$ (**e**,**f**) limit the choice of $\textbf{C}$ to close near maximum cooling $\textbf{C}=0$ (black dots) to maintain a negligible back-action regime for the same $t_{tot}$.

**Figure 4:**
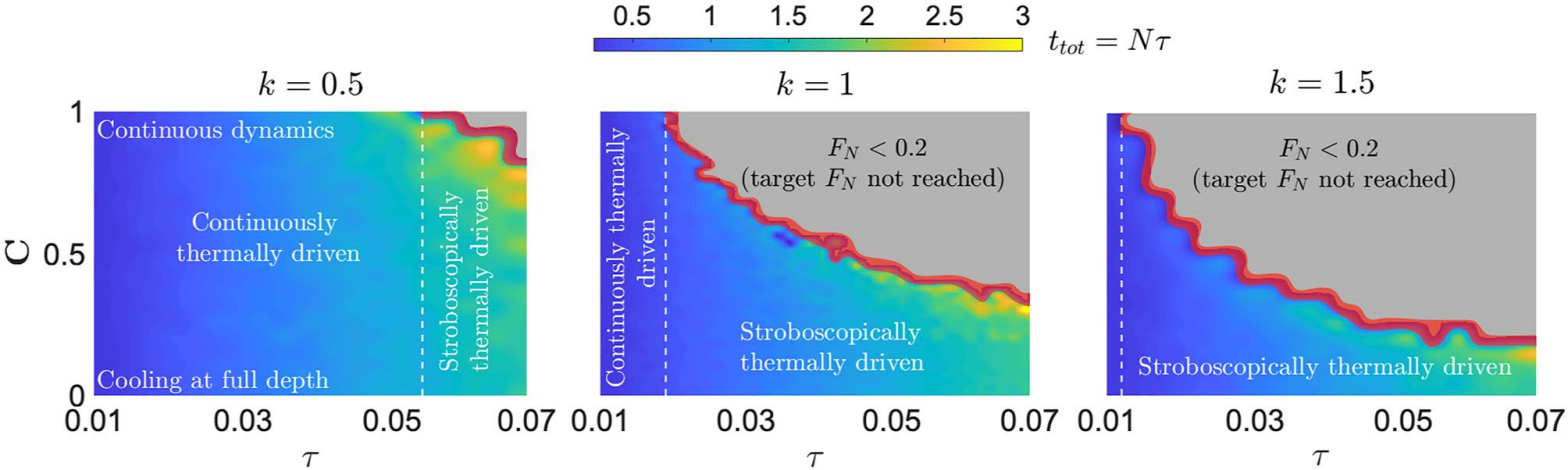
Optimisation of the stroboscopic cooling at different strengths of the interaction *k*. The colorbar $t_{tot}=N\tau$ indicates the total time required to achieve the target noise factor $F _N=0.2$ for a given $\textbf{C}$ and $\tau$. The grey region consists of such dynamics, dominated by the back-action, where the achieved noise factor is below the target $F_N<0.2$, i.e., the $\textrm{SNR}_x(t_{tot})$ drops before reaching the $F_N=0.2$. As the interaction strength *k* increases (left to right) the *Stroboscopic thermally driven* dynamics is advantageous. In fact, the back-action dominating area (grey), appearing after the threshold values of $\tau$ (withe dashed line) hinders the *Continuously thermally driven* dynamics to reach the target noise factor. Note how an increase in *k* noticeably modifies the $\tau$-threshold value. The green/yellow areas represent the subset of values of the parameter space where the back-action affects the dynamics (the $\textrm{SNR}_x$ diverges from the $\sqrt{N}$ scaling) while allowing $F _N=0.2$ to be reached at the cost of an increased total time $t_{tot}$. Lastly, the red area is the *uncertain* region where the finite sampling of the numerical simulation creates a rough transition between the grey and the blue/yellow area. Consequently, it represents the least optimal region to operate the stroboscopic control. Differently to other plots, here $T_x=0$ is used.

A change in the initial uncertainty $\sigma _{x_0}^2$, environmental noise $T_x$, or the single cycle time $\tau$ can increase the back-action and ultimately decrease the $\textrm{SNR}_x(t_{tot})$ for finite repetitions. As the full dynamics of Eq. (4) is too complex to be solved analytically, the effect of the back-action is observed in Figs. 3 and 4 as a deviation from the scaling with the number of cooling events *N* of Eq. (5).

Firstly, the stroboscopic control is tested under larger initial position uncertainties $\sigma _{x_0}^2$ in Fig. 2 (panels a,c,e) with fixed $\tau =0.1, T_x=0$. An increasing initial variance is detrimental for the increase of the signal-to-noise ratio over *N*. While deep cooling $\textbf{C} \rightarrow 0$ (black) always outperforms the continuous dynamics with no confinement and cooling $\textbf{C} \rightarrow 1$ (red, blue) for small initial variance $\sigma _{x_0}^2$ (panels a,c), it is not the case for larger $\sigma _{x_0}^2$ (panel e). However, at larger repetitions (from $N\approx 15$) the deep cooling still overcomes the growth of the continuous dynamics (red, blue). Note, the total time of stroboscopic control $t_{tot}=\tau N$, before the drop of the signal-to-noise ratio, is conserved as $\sigma _{x_0}^2$ increases, and only the scaling is modified.

Secondly, the robustness is tested against different temperatures of the system’s environment $T_x$ in Fig. 2 (panels b,d,f), with fixed $\tau =0.1, \sigma _{x_0}^2=0$. Advantageously, a large cooling depth $\textbf{C}$ (black dots) helps to minimise the deviation from the ideal case (solid black line) that is maximising the $\textrm{SNR}$. Note, a large change in $T_x$ (panels f) does not change appreciably the result from the pure deterministic $T_x=0$ case (panels b).

Lastly, the requirement on single cycle times $\tau$ is analysed, with fixed $T_x=0.1,\sigma _{x_0}^2=0$ in Fig. 3. Panels (a,c,e) show the linear *N* scaling for mean values of *x*, while panels (b,d,f) the $\sqrt{N}$ scaling of the signal-to-noise ratio. For a larger time $\tau$ between two cooling events, an intensive cooling $\textbf{C}$ (black dots) close to maximum depth helps to minimise the otherwise growing back-action. Notice how a small change in $\tau$ (panels b,d) can appreciably change the requirements on the cooling depth $\textbf{C}$. For $T_x \rightarrow 0$, larger times $\tau$ between cooling events and shallower depth of cooling $\textbf{C}\rightarrow 1$ are allowed.

### Noise factor

The inherent costs of the stroboscopic control lie in the depth of cooling $\textbf{C}$ and the shortening of the single-cycle time $\tau$ required to maximise the output $\textrm{SNR}_x(t_{tot})$. To better visualise the costs, we introduce the noise factor $F (t_{tot})=\textrm{SNR}_{x(\tau )}/\textrm{SNR}_x(t_{tot})$^56^ defined as the ratio of the single-cycle signal-to-noise ratio $\textrm{SNR}_{x(\tau )}$, and the signal-to-noise ratio of the Nth-cycle $\textrm{SNR}_x(t_{tot})$ obtained after the stroboscopic control, where $t_{tot}=N\tau$ is the total time of the dynamics. The noise factor quantifies the performance of the stroboscopic cooling. The lower the $F _N$ value, the higher the performance. Fruitfully, for the back-action-less regime at $T_x,\sigma _{x_0}^2=0$, the stroboscopic evolution of Eq. (4) simplifies the signal-to-noise ratio $\textrm{SNR}_x(t_{tot})=\sqrt{N}\textrm{SNR}_{x(\tau )}$ to that of Eq. (5). In such limit, the noise factor is solely dependent on the number of cooling events

6 $$\begin{aligned} F _N=\frac{\textrm{SNR}_{x(\tau )}}{\textrm{SNR}_x(t_{tot})} = \frac{\textrm{SNR}_{x(\tau )}}{\sqrt{N} \textrm{SNR}_{x(\tau )}} = \frac{1}{\sqrt{N}}. \end{aligned}$$

To outline the expected gain on the signal-to-noise ratio, the target noise factor of $F _N=0.2$ is fixed. From Eq. (6), such noise factor can be reached with $N_{target}=25$ cooling events. However, a full simulation, with back-action included, achieves $F _N=0.2$ for generally different *N*. Fig. 4 analyses the costs required to achieve the target noise factor for various $\textbf{C}$, and $\tau$. The choice of $F _N=0.2$ is such that the saturation of the $\sqrt{N}$ scaling of the signal-to-noise ratio of Fig. 1 (panel (d)) is maximised, i.e., minimise the distance between black dots and solid line.

For a given $\textbf{C}$, and $\tau$, the colorbar of Fig. 4 displays the total time $t_{tot}=N\tau$ required to reach $F _N=0.2$, ideally being as small as possible for optimal performance. When the coupling strength *k* is comparable to the damping rate $\Gamma$, Fig. (4, centre), for times $\tau \lessapprox 0.02$ the target noise figure $F_N=0.2$ can be reached even by the continuously thermally driven dynamics $\textbf{C}=1$. It therefore unveils a regime where the stroboscopic control is not needed as the cooling to $\textbf{C}<1$ is more costly. However, for increasing times $\tau \gtrapprox 0.02$, the back-action hinders the *continuously thermally driven* dynamics from reaching the target noise factor $F_N=0.2$. The required depth of cooling increases $\textbf{C}\rightarrow 0$, and the stroboscopic control proves to be advantageous. The latter can always reach the target $F_N$, however, at the cost of longer overall duration $t_{tot}$ as visible in Fig. 4. The region to employ the stroboscopic control can change with the strength of the interaction *k*. In the weak coupling regime $k<1$ (Fig. 4 left) the grey area becomes smaller compared to that obtained at $k=1$, while the region of the stroboscopic control moves to larger times $\tau$. In contrast, when in the strong coupling regime $k>1$ (Fig. 4 right) the grey area widens to smaller times $\tau$, thus broadening the region of the stroboscopic control to $\tau \rightarrow 0$.

In all cases, when approaching the grey area, the finite sampling of the numerical simulations creates rough transitions. They are labelled as *uncertain* regions (red), and are obtained by scanning the numerical data and flagging the pixels where the sudden transitions between $t_{tot}$ appears. Note, this scan is only performed at the edge of the grey area.

To saturate the scaling and minimise the back-action the costs of the stroboscopic control, namely depth of cooling $\textbf{C}$ and single cycle time $\tau$, have been introduced in Fig. 4 for the ideal $T_x,\sigma _{x_0}^2=0$ case. In a more realistic scenario, $\mathbf {\theta _0}, \mathbf {\theta _E} \ne 0$, a further minimisation is required. An optimal control of the environmental temperature $T_x$ over the stroboscopic control can be implemented to further delay the back-action. However, this is beyond the scope of this work.

## Conclusions

Unstable asymmetrical systems can turn fluctuations into directional motion. However, the limitation upon observing the effect in a one-dimensional nonlinear system (such as the cubic potential^29,51,52^) arises from the rapidly increasing noise from that unstable behaviour. A combination of a thermal bias and stroboscopic cooling can overcome the limitation. Advantageously, it does not require any challenging higher-order nonlinearities.

To observe the nonlinear effect of thermally-induced motion through asymmetrical nonlinear interactions, i.e., $xy^2$, the natural choice of over-damped dynamics eases the experimental measurement of such effect, as previously operated in^29,51^. The stroboscopic control with cooling of the drive variable *y* allows a sensible enhancement of the signal-to-noise ratio (as evidenced in Fig. 1) beyond the capabilities of a one-dimensional cubic potential^51^. In the stroboscopic control, the limitation given by the increasing noise in *x* can be efficiently controlled through cooling of the driving variable *y*.

An alternative control of the back-action relies on a non-autonomous control (feedback) measuring *only* the position of the drive variable *y*. By manipulating its dynamics with a linear restoring force, the back-action can be further withstood and the signal-to-noise ratio enhanced. Doing so it relaxes the conditions upon $\textbf{C}$, and $\tau$. The possibilities recently demonstrated on the control of mechanical motion with nano-seconds sampling^19^, pave the way for testing this strategy in levitodynamics experiments. Alternatively, the asymmetric nonlinear potential can be fully engineered by non-conservative forces, where the back-action is completely removed leaving only the positive asymmetrical interaction to be exploited^57^.

Another approach requires different nontrivial geometries to be implemented. Their main focus is to improve the scaling of the stroboscopic control. An interesting candidate, showing positive improvements, includes $V(x,y)\propto xy^2 + \lambda x^2y$. Numerical simulations of this model show a linear scaling of the signal-to-noise ratio $N\textrm{SNR}_{x(\tau )}$ as opposed to $\sqrt{N}\textrm{SNR}_{x(\tau )}$ in the basic model discussed in this paper. It can extend the nonlinear stroboscopic control without a need for higher-order nonlinearity.

In the limit of low pressure, the position of the system’s variable *x* barely moves around its initial conditions $\langle x_0 \rangle$. However, the main nonlinear effect is translated to the instantaneous velocity $\dot{x}$ ^52^. In the stroboscopic control, while the cooling of the driver *y* is implemented by feedback control to the desired $\textbf{C}$, the noise from the driver *y* induces the motion in the mean instantaneous velocity $\langle \dot{x} \rangle$. Such a low pressure regime allows for a better control of external noises $T_x>0$, and larger repetitions *N* before the back-action dominates the entire evolution, as compared to the high pressure regime. The stroboscopic control realises a large signal-to-noise ratio in both position $\textrm{SNR}_{x}(t_{tot})$ and velocity $\textrm{SNR}_{\dot{x}}(t_{tot})$, climbing above the critical value $\textrm{SNR}_x=1$ with only $2\tau$ repetitions. However, such signal-to-noise ratio improvement comes with a smaller shift of both mean position $\langle x(t_{tot}) \rangle$ and velocity $\langle \dot{x}(t_{tot}) \rangle$. Note that the scalings of Eq. 5 are conserved for all quantities, and that in the limit of large damping, the results of this paper are retrieved.

When the overall dynamics approaches the unitary one, in the deeply quantum limit, the thermal bath of the driving variable *y* can be stroboscopically substituted by a quantum noise to ultimately observe thermally induced coherent quantum phenomena on the system *x*^58^.

Advantageously, experimental realisations of asymmetrical nonlinearities similar to $kxy^2$, have been recently achieved in trapped ions^59,60^, SNAIL^61,62^ and superconducting circuits^63^ via trilinear interactions. Levitodynamics has already shown instances where such asymmetrical interactions can be in principle observed^41,57,64^, allowing for immediate experimental verification.

## Methods

### Calculation of the higher order correlation $\langle y(t')^2y(t'')^2 \rangle$

In this section we show the full derivation of the higher order correlation $\langle y^2(t') y^2(t'') \rangle$ and the calculation of the second moment of particle position $\langle x(t)^2\rangle$ starting from Eq. (1), to recover the result of the standard deviation $\sigma _x(t)$ of Eq. (2). For ease of presentation, throughout this methods section, the multiple integral $\int _0^t \int _0^t \cdots \int _0^t dt_1\,dt_2\, \cdots \,dt_N$ will be simplified as $\int _0^t dt_1\,dt_2\, \cdots \,dt_N$^65^.

Firstly, let us recall few important equalities that will be called throughout:

7 $$\begin{aligned} \langle W_{12}' \rangle&= \int _0^{t'} \langle \xi (t_1)\xi (t_2) \rangle dt_1\,dt_2,\nonumber \\ \langle W_{12}'' \rangle&= \int _0^{t''} \langle \xi (t_1)\xi (t_2) \rangle dt_1\,dt_2,\nonumber \\ \langle W_1'W_1'' \rangle&= \int _0^{t'}\int _0^{t''} \langle \xi (t_1)\xi (t_2) \rangle dt_1\,dt_2\nonumber \\ \langle W_{12}'W_{12}'' \rangle&= \int _0^{t'}\int _0^{t''} \langle \xi (t_1)\xi (t_2)\xi (t_3)\xi (t_4) \rangle dt_1\,dt_2\,dt_3\,dt_4=\nonumber \\&=\int _0^{t'}\int _0^{t''} \langle \xi (t_1) \xi (t_2) \rangle \langle \xi (t_3) \xi (t_4) \rangle + \langle \xi (t_1) \xi (t_4) \rangle \langle \xi (t_2) \xi (t_3) \rangle + \langle \xi (t_1) \xi (t_3) \rangle \langle \xi (t_2) \xi (t_4) \rangle dt_1\,dt_2\,dt_3\,dt_4, \end{aligned}$$

where the following Wick contraction has been used^65^

$$\begin{aligned} \langle \xi _i\xi _j \xi _k \xi _l \rangle = \langle \xi _i\xi _j \rangle \langle \xi _k\xi _l \rangle +\langle \xi _i\xi _l \rangle \langle \xi _j\xi _k \rangle +\langle \xi _i\xi _k \rangle \langle \xi _j\xi _l \rangle, \end{aligned}$$

simplified by noting that when the set of indices $i,j,\ldots, k,l$ contains an odd number of elements, $\langle x_i x_j \cdots x_k x_l \rangle$ vanishes trivially.

Secondly, using the property of Gaussian white Markovian noise, i.e., $\langle \xi _i\xi _j \rangle = \delta (t_i-t_j)=\delta _{ij}$^53^, the integrals of Eq. (7) can be computed, leading to

8 $$\begin{aligned} \langle W_{12}' \rangle&= \int _0^{t'} \delta _{12} dt_1\,dt_2 = t',\nonumber \\ \langle W_{12}'' \rangle&= \int _0^{t''} \delta _{12} dt_1\,dt_2= t'',\nonumber \\ \langle W_1'W_1'' \rangle&= \int _0^{t'}\int _0^{t''} \delta _{12} dt_1\,dt_2 = t''+(t'-t'') \mathcal {H}(t''-t'),\nonumber \\ \langle W_{12}'W_{12}'' \rangle&= \int _0^{t'} \int _0^{t''} \delta _{12}\delta _{34} + \delta _{14}\delta _{23} + \delta _{13}\delta _{24} dt_1\,dt_2\,dt_3\,dt_4 = \nonumber \\&= t't'' + t''^2t'^2\mathcal {H}(t''-t')-t''^2\mathcal {H}(t''-t') +t''^2t'^2\mathcal {H}(t''-t')-t''^2\mathcal {H}(t''-t'), \end{aligned}$$

where $\mathcal {H}(\tau )$ is the Heaviside step function^66^.

From the back-action-less (without the $-kxy$ term) evolution of the drive variable *y* of Eq. (1), one obtains $y(t')=y_0 +\sqrt{2T_y}\int _0^{t'}\xi (t_1)dt_1$, leading to $y(t')^2=y_0^2 + 2 y_0 \sqrt{2T_y}\int _0^{t'} \xi (t_1)dt_1 + 2T_y \int _0^{t'} \xi (t_1)\xi (t_2)dt_1\,dt_2$. For simplicity we assumed $k_B=1,m=1,\Gamma =1$. In the extreme regime of deterministic evolution of particle position *x* of Eq. (1), i.e., $T_x=0, x_0=0$, the second moment $\langle x^2 \rangle = k^2 \int _0^t \langle y(t')^2y(t'')^2 \rangle dt' dt''$ depends solely on the integral of the higher order correlation $\langle y(t')^2y(t'')^2 \rangle$. By substituting the expression of $y(t')^2$, and $y(t'')^2$ in the correlation term and expanding, assuming $\langle y_0 \rangle =0$ and recalling the equality $\sigma _{y_0}^2=T_{0,y}$ one obtains

9 $$\begin{aligned} \langle y(t')^2y(t'')^2 \rangle = 3 T_{0,y}^2 + 2T_y T_{0,y} \left( \langle W_{12}'' \rangle + 4\langle W_1' W_1'' \rangle + \langle W_{12}' \rangle \right) +4 T_y^2 \langle W_{12}' W_{12}'' \rangle . \end{aligned}$$

Substituting the result of Eq. (8) into Eq. (9), the integral of the high order correlation can be computed $\int _0^t \langle y(t')^2y(t'')^2 \rangle dt' dt'' = 3 T_{0,y}^2 t^2 +14 T_{0,y}T_y t^3/3 + 7T_y^2 t^4/3$, and the second moment of particle position $\langle x^2 \rangle$ becomes

10 $$\begin{aligned} \langle x^2 \rangle =k^2\int _0^t \langle y(t')^2y(t'')^2 \rangle dt' dt'' = k^2T_y^2 t^4 \left[ 3 \frac{\textbf{C}^2}{t^2} + \frac{14 \textbf{C}}{3 t} + \frac{7}{3} \right], \qquad \textbf{C}=\frac{T_{0,y}}{T_y}, \end{aligned}$$

thus leading to the standard deviation $\sigma _x(t)=(2/\sqrt{3})k T_y t^2 \sqrt{3 \textbf{C}^2/t^2 + 14\textbf{C}/3 t + 7/3}$ of Eq. (2).

### Stroboscopic evolution

In this section we discuss the stroboscopic evolution with cooling of the drive variable *y*, to recover the results for the moments of particle position $\langle x(t_{tot}) \rangle,\sigma _x(t_{tot})$ of Eq. (4), and the ideal control of the back-action-less regime of Eq. (5).

The stroboscopic evolution is obtained by periodically cooling the continuous motion of the drive variable *y*, by a series of fast cooling events. We thus describe the motion for a finite time $\tau$ (time of the single period) by virtue of Eq. (1), in the approximation of negligible back-action, as follows

11 $$\begin{aligned} x(\tau )&= x_0 - k \int _0^{\tau } y^2(t')dt' + \sqrt{\frac{2k_B T_x}{\Gamma }} \int _0^{\tau } \xi _x(t')dt', \end{aligned}$$

12 $$\begin{aligned} y(t')&\approx y_0 + \sqrt{\frac{2k_B T_y}{\Gamma }}\int _0^{t'} \xi _y(t_1)dt_1, \end{aligned}$$

where at initial time $t=0$ the trajectories $x_0,y_0$ are obtained by a zero mean Gaussian distribution with variances $\sigma ^2_{x_0}=k_B T_{0,x}/m \omega _{0,x}^2$, $\sigma ^2_{y_0}=k_B T_{0,y}/m \omega _{0,y}^2$. After the first cycle time $t=\tau$, the drive variable *y* is cooled by a depth of cooling $\textbf{C}=T_{0,y}/T_y$, thus restoring its uncertainty to its initial variance $\sigma _{y_0}^2$, while the evolution of *x* remains continuous. The subsequent cycle runs from $t=[0, 2\tau ]$, and at $t=\tau$ the drive variable *y* is restarted to a new initial fluctuating $y_0$, characterised by a vanishing first moment $\langle y_0 \rangle =0$ and a variance $\sigma _{y_0}^2$ dictated by $\textbf{C}$, statistically independent to both *x* and the Langevin forces, while the dynamics in the position *x* continues its evolution to $t=2\tau$. The latter can be described as follows

$$\begin{aligned} x(2\tau )&= x_0 - k \left[ \int _0^{\tau } y^2_1(t') dt' + \int _{\tau }^{2\tau } y^2_2(t')dt'\right] + \sqrt{\frac{2k_B T_x}{\Gamma }} \int _0^{2\tau } \xi _x(t')dt',\nonumber \\ y_1(t')&\approx y_{0,1} + \sqrt{\frac{2k_B T_y}{\Gamma }}\int _0^{t'} \xi _y(t_1)dt_1.\nonumber \\ y_2(t')&\approx y_{0,2} + \sqrt{\frac{2k_B T_y}{\Gamma }}\int _0^{t'} \xi _y(t_1)dt_1, \end{aligned}$$

Since the time interval between the two cooling events $\tau -0, 2\tau -\tau =\tau$ are constant, the sum of the two integrals can be simplified to

13 $$\begin{aligned} \begin{aligned} x(2\tau )&= x_0 - k \left[ \int _0^{\tau } y^2_1(t') dt' + \int _0^{\tau } y^2_2(t') dt' \right] + \sqrt{\frac{2k_B T_x}{\Gamma }} \int _0^{2\tau } \xi _x(t')dt',\\ y_n(t')&\approx y_{0,n} + \sqrt{\frac{2k_B T_y}{\Gamma }}\int _0^{t'} \xi _y(t_1)dt_1, \end{aligned} \end{aligned}$$

where the index $n=1,2$ indicates the trajectories of the drive variable $y_1$ at the first stroboscopic step, and $y_2$ at the second stroboscopic step. From Eq. (13), the first moment of particle position $x(2\tau )$, under the assumption of $\langle x_0 \rangle =0$ can be calculated to be

14 $$\begin{aligned} \langle x(2\tau ) \rangle = - k \left[ \int _0^{\tau } \langle y_1^2(t')\rangle dt' + \int _{0}^{\tau } \langle y_2^2(t')\rangle dt' \right] . \end{aligned}$$

Simultaneously, the second moment of position $\langle x^2(2\tau ) \rangle$ approaches

15 $$\begin{aligned} \begin{aligned} \langle x(2\tau )^2 \rangle&=\langle x_0^2 \rangle +k^2 \left( \iint _0^{\tau } \langle y^2_1(t') y^2_1(t'') \rangle dt'\,dt'' + \iint _{0}^{\tau } \langle y^2_2(t') y^2_2(t'') \rangle dt'\,dt'' + 2 \iint _0^{\tau }\langle y^2_1(t') y^2_2(t'') \rangle dt'\,dt''\right) +\\&\quad +\frac{2k_B T_x}{\Gamma }\iint _0^{t_{tot}} \langle \xi _x(t')\xi _x(t'') \rangle dt'\,dt'', \end{aligned} \end{aligned}$$

unveiling the extra correlation term of the *y*-trajectories between different cooling events $\langle y^2_1(t') y^2_2(t'') \rangle$. Note that since the *y*-trajectories of the two stroboscopic steps are each described by independent free diffusion processes, and hence are uncorrelated, the cross correlation $\langle y^2_1(t') y^2_2(t'') \rangle = \langle y^2_1(t')\rangle \langle y^2_2(t') \rangle$ decomposes into the product of their mean values, resulting in $\langle y^2_n(t') \rangle = \sigma _{y_0}^2 + 2k_BT_y t'/\Gamma$ as obtained above Eq. (2).

From Eqs. (14) and (15), the standard deviation of position $\sigma _x(2\tau )=\sqrt{\langle x(2\tau )^2 \rangle - \langle x(2\tau ) \rangle ^2}$ approaches

16 $$\begin{aligned} \sigma _x(2\tau )=\sqrt{\sigma _{x_0}^2+ k^2 \left( \iint _0^{\tau } \textbf{K}_{1,1}(t',t'') dt'\,dt'' + \iint _{0}^{\tau } \textbf{K}_{2,2}(t',t'') dt'\,dt''\right) + \frac{4 k_B T_x \tau }{\Gamma }}, \end{aligned}$$

where $\textbf{K}_{n,n}(t',t'')= \langle y^2_n(t') y^2_n(t'') \rangle -\langle y^2_n(t') \rangle \langle y^2_n(t') \rangle$ is the auto covariance of the drive variable $y^2_n$ acting on the position *x*. Remember that the cross covariance $\textbf{K}_{n,m}=\langle y^2_1(t') y^2_2(t'') \rangle - \langle y^2_1(t')\rangle \langle y^2_2(t') \rangle$ vanished due to the uncorrelated nature of the *y*-trajectories between the two stroboscopic steps. In the limit of $T_x=0$ of an initially fully cooled position $\sigma _{x_0}^2=0$, it can be shown that each stroboscopic step generates the same auto correlation $\langle y^2_n(t')\rangle$ from Eq. (14) and auto covariance $\textbf{K}_{n,n}(t',t'')$ from Eq. (16), thus leading to the following moments

17 $$\begin{aligned} \begin{aligned} \langle x(2\tau ) \rangle&=-2 k T_y\left( \frac{\textbf{C}}{t}+1\right) t^2 = 2 \langle x(\tau ) \rangle,\\ \sigma _x(2\tau )&= \frac{2\sqrt{2}}{\sqrt{3}}k T_y \left( 3 \frac{\textbf{C}^2}{t^2} + \frac{14\textbf{C}}{3 t} + \frac{7}{3} \right) ^{\frac{1}{2}}t^2 =\sqrt{2} \sigma _x(\tau ),\\ \textrm{SNR}_x(2\tau )&= \frac{| \langle x(2\tau ) \rangle |}{\sigma _x(2\tau )} = \sqrt{\frac{3}{2}} \frac{\frac{\textbf{C}}{t} + 1}{\sqrt{\frac{3\textbf{C}^2}{2t^2} +2\frac{\textbf{C}}{t} +1}}=\sqrt{2} \textrm{SNR}_x(\tau ), \end{aligned} \end{aligned}$$

where the enhancement of the signal-to-noise ratio by a factor of $\sqrt{2}$ is due to the stroboscopic control.

For many cycles $t=[0,N\tau ]$, without loss of generality, one can rewrite Eq. (13) as

18 $$\begin{aligned} \begin{aligned} x(t_{tot})&= x_0 - k \sum _{n=0}^N \left[ \int _0^{\tau } y^2_n(t') dt' \right] + \sqrt{\frac{2k_B T_x}{\Gamma }} \int _0^{t_{tot}} \xi _x(t')dt',\\ y_n(t')&\approx y_{0,n} + \sqrt{\frac{2k_B T_y}{\Gamma }}\int _0^{t'} \xi _y(t_1)dt_1, \end{aligned} \end{aligned}$$

where $t_{tot}=N\tau$ is the total time of the stroboscopic evolution, and $y_n$ describes the dynamics of the drive variable between two cooling events.

Similarly, the moments of particle position *x* of Eqs. (14) and (16) can be written as

19 $$\begin{aligned} \begin{aligned} \langle x(t_{tot}) \rangle&= - k \sum _{n=0}^N \int _0^{\tau }\langle y^2_n(t')\rangle dt',\\ \sigma _x(t_{tot})&=\sqrt{\sigma _{x_0}^2+ k^2\sum _{n=0}^N \iint _0^{\tau } \textbf{K}_{n,n}(t',t'')dt' dt'' + \frac{2 k_B T_x t_{tot}}{\Gamma }}, \end{aligned} \end{aligned}$$

recovering the result of Eq. (4).

Note that the terms $\sum _{n=0}^N \langle y^2_n(t')\rangle = N \langle y^2_n(t')\rangle$, and $\sum _{n=0}^N \textbf{K}_{n,n}(t',t'')= N \textbf{K}_{n,n}(t',t'')$ from Eq. (19) increase by a constant amount, and in the limit of $T_x,\sigma _{x_0}^2=0$ without the back-action, the scaling on the number of stroboscopic steps *N* arises as

20 $$\begin{aligned} \begin{aligned} \langle x(t_{tot}) \rangle&=-N k T_y\left( \frac{\textbf{C}}{t}+1\right) t^2 = N \langle x(\tau ) \rangle,\\ \sigma _x(t_{tot})&= \frac{2\sqrt{N}}{\sqrt{3}}k T_y \left( 3 \frac{\textbf{C}^2}{t^2} + \frac{14\textbf{C}}{3 t} + \frac{7}{3} \right) ^{\frac{1}{2}}t^2 =\sqrt{N} \sigma _x(\tau ),\\ \textrm{SNR}_x(t_{tot})&= \frac{| \langle x(t_{tot}) \rangle |}{\sigma _x(t_{tot})} = \sqrt{N}{\frac{\sqrt{3}}{2}} \frac{\frac{\textbf{C}}{t} + 1}{\sqrt{\frac{3\textbf{C}^2}{2t^2} +2\frac{\textbf{C}}{t} +1}}=\sqrt{N} \textrm{SNR}_x(\tau ), \end{aligned} \end{aligned}$$

retrieving the result of Eq. (5).

### Numerical simulation methods

To numerically simulate the Langevin dynamics described in Eq. (1), the corresponding stochastic differential equation

21 $$\begin{aligned} dx = - k y^2 dt + \sqrt{\frac{2k_B T_x}{\Gamma _x}}\xi _x(t) dt,\qquad dy = -2 k x y dt +\sqrt{\frac{2k_B T_y}{\Gamma _y}}\xi _y(t) dt, \end{aligned}$$

is integrated in time with the Euler-Maruyama algorithm, leading to

22 $$\begin{aligned} x(t) =x_0 - k \int _0^t y^2(t') dt' + \sqrt{\frac{2k_B T_x}{\Gamma _x}}\int _0^t \xi _x(t') dt',\qquad y(t')= y_0 -2 k \int _0^{t'} x(t_1) y(t_1) dt_1 +\sqrt{\frac{2k_B T_y}{\Gamma _y}} \int _0^{t'} \xi _y(t_1) dt_1. \end{aligned}$$

**Figure 5:**
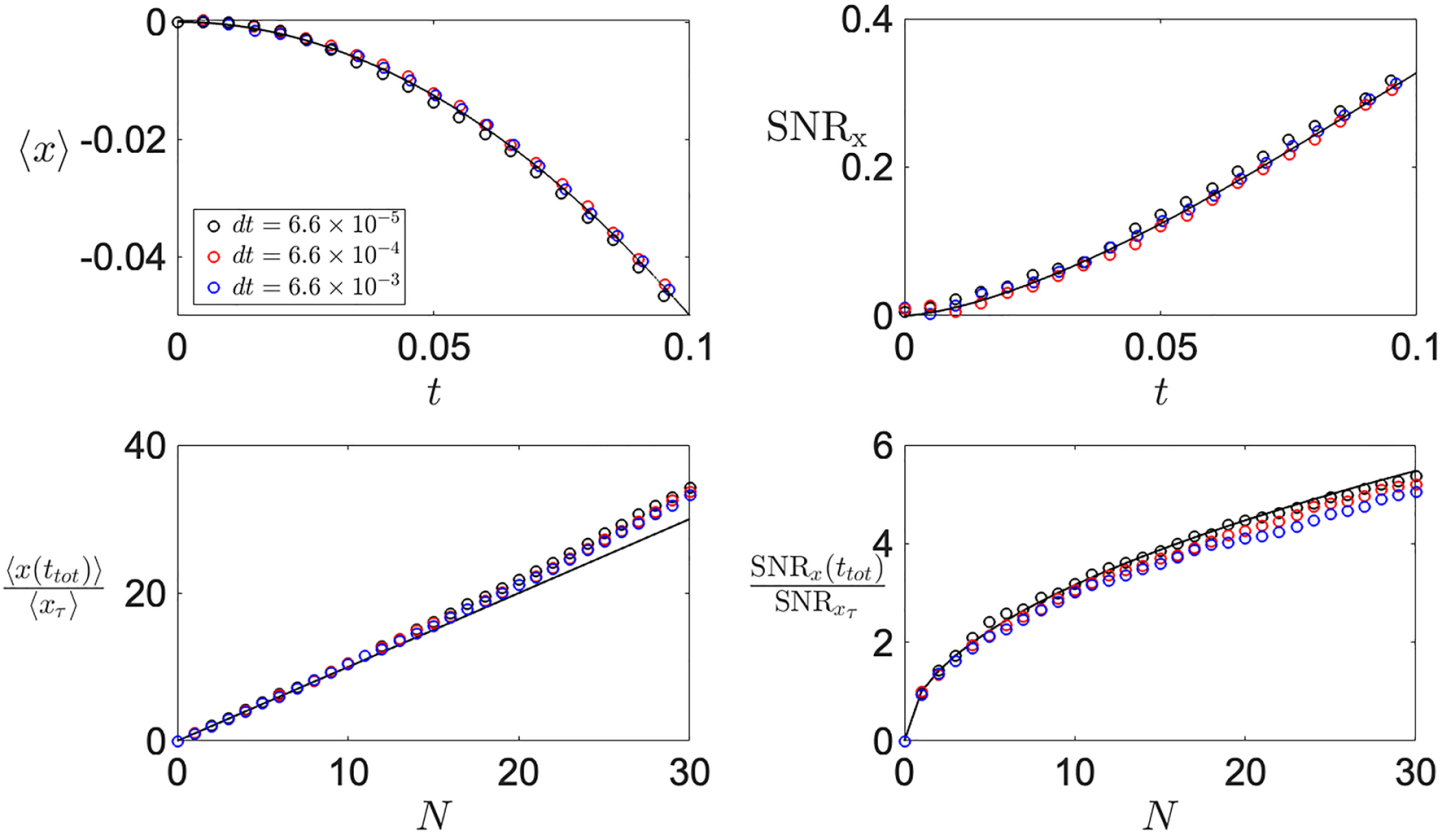
Time-step convergence of numerical simulations. *Top*: Single cycle dynamics of mean position (left) and signal-to-noise ratio (right) shows a fast convergence of time step requirements to nicely observe the thermally-induced effect. *Bottom*: Stroboscopic control of mean position (left) and signal-to-noise ratio (right) shows a fast convergence of time-step requirements for small number of repetitions. However, with increasing *N*, a smaller time-step (black dots) is required, given by the back-action becoming more relevant in the dynamics.

While is important to utilise the proper time-step, in Fig. 5 it can be seen that for the single cycle evolution (top) the window of usable time-steps *dt* allows for two order of magnitude that won’t hinder the observation of the thermally induced effect with increasing signal-to-noise ratio. However, when simulating the stroboscopic control (Fig. 5, bottom), the choice of the time-step *dt* becomes more relevant for larger repetitions where the back-action starts to affect the motion (see panel d, blue versus black dots).

**Figure 6:**
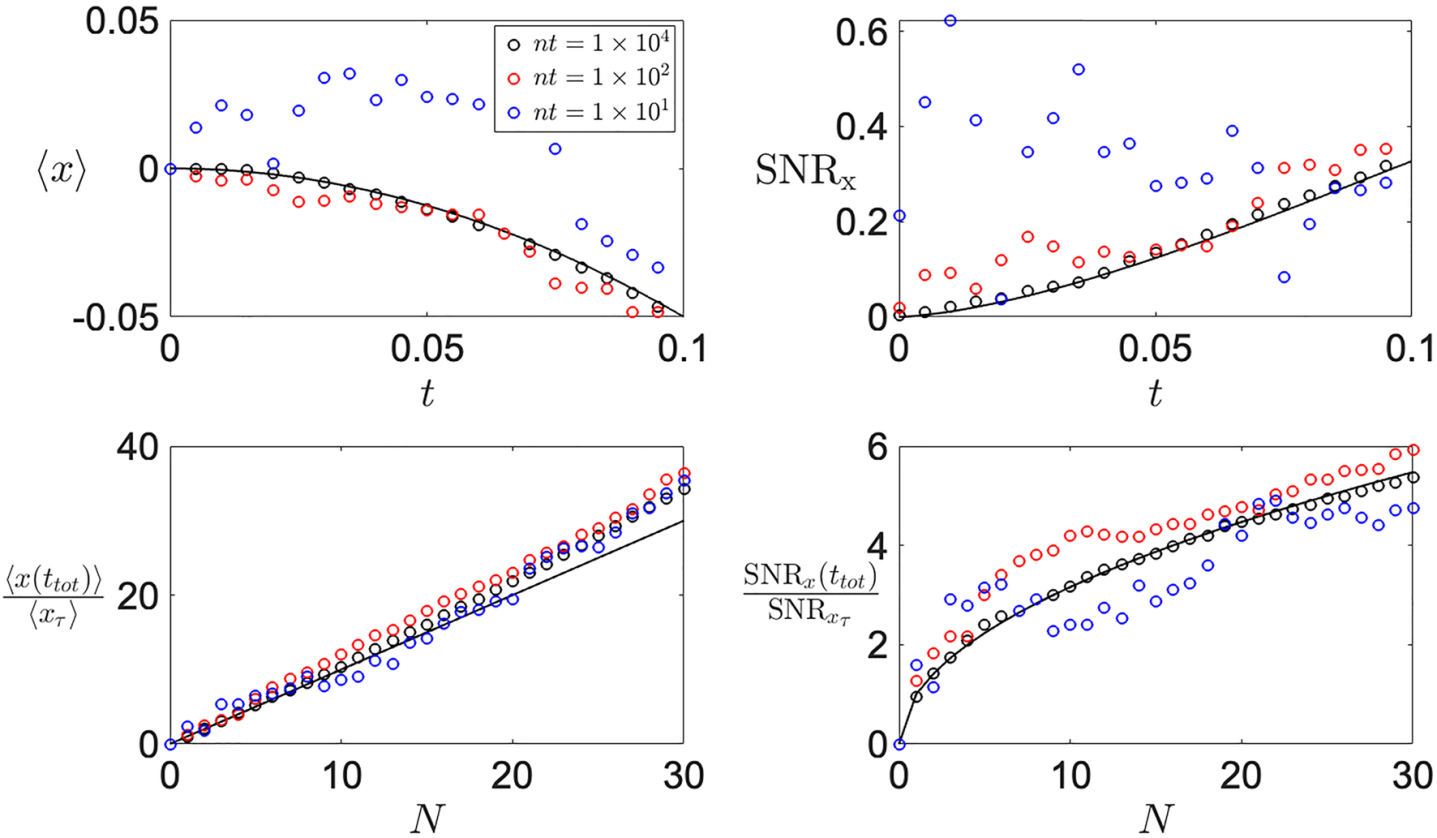
Ensemble size *nt* convergence of numerical simulation. *Top*: Single cycle dynamics of mean position (left) and signal-to-noise ratio (right) show how small ensemble size $nt=10^1$ (blue) results in a large fluctuation, hindering the observation of the thermally-induced effect. While an increase of an order of magnitude $nt=10^2$ (red) greatly improves the observation of the effect, an ensemble size $nt=10^4$ (black) minimises the fluctuation for better comparison with analytical results. *Bottom*: Stroboscopic control of mean position (left) and signal-to-noise ratio (right) show a greater sensitivity to the ensemble size. An ensemble size $nt=10^2$ (red) almost always overestimates the signal-to-noise ratio (right), while smaller ensemble size fluctuates more violently (blue). As for the single cycle dynamics, an ensemble size of $nt=10^4$ (black) minimises the fluctuations and nicely estimates the relevant quantities.

In contrast, the choice upon the ensemble size is of much more importance. The mean position of the system $\langle x(t) \rangle = -k\int _0^t \langle y^2(t') \rangle dt'$ is dictated by the integral of the autocorrelation $\langle y^2(t') \rangle$, and as shown in Fig. 6 (red,blue), a small ensemble size ($nt< 10^4$) leads to larger fluctuations in the simulated data. Ultimately, these large fluctuations negatively impact the signal-to-noise ratio of both the single cycle (top) and stroboscopic control (bottom), which become even more relevant when back-action dominates.

## Data Availability

The data that support the findings of this study are available from the corresponding author upon reasonable request.
